# A Sequence Identification Measurement Model to Investigate the Implicit Learning of Metrical Temporal Patterns

**DOI:** 10.1371/journal.pone.0075163

**Published:** 2013-09-25

**Authors:** Benjamin G. Schultz, Catherine J. Stevens, Peter E. Keller, Barbara Tillmann

**Affiliations:** 1 MARCS Institute, University of Western Sydney, Sydney, New South Wales, Australia; 2 Lyon Neuroscience Research Center, Team Auditory Cognition and Psychoacoustics, Centre National de la Recherche Scientifique, Unité Mixtes de Recherche 5292, Institut National de la Santé et de la Recherche Médicale U1028, Université Lyon, Lyon, Rhône-Alpes, France; 3 Max Planck Institute for Human Cognitive and Brain Sciences, Leipzig, Saxony, Germany; UNLV, United States of America

## Abstract

Implicit learning (IL) occurs unconsciously and without intention. Perceptual fluency is the ease of processing elicited by previous exposure to a stimulus. It has been assumed that perceptual fluency is associated with IL. However, the role of perceptual fluency following IL has not been investigated in temporal pattern learning. Two experiments by Schultz, Stevens, Keller, and Tillmann demonstrated the IL of auditory temporal patterns using a serial reaction-time task and a generation task based on the process dissociation procedure. The generation task demonstrated that learning was implicit in both experiments via motor fluency, that is, the inability to suppress learned information. With the aim to disentangle conscious and unconscious processes, we analyze unreported recognition data associated with the Schultz et al. experiments using the *sequence identification measurement model*. The model assumes that perceptual fluency reflects unconscious processes and IL. For Experiment 1, the model indicated that conscious and unconscious processes contributed to recognition of temporal patterns, but that unconscious processes had a greater influence on recognition than conscious processes. In the model implementation of Experiment 2, there was equal contribution of conscious and unconscious processes in the recognition of temporal patterns. As Schultz et al. demonstrated IL in both experiments using a generation task, and the conditions reported here in Experiments 1 and 2 were identical, two explanations are offered for the discrepancy in model and behavioral results based on the two tasks: 1) perceptual fluency may not be necessary to infer IL, or 2) conscious control over implicitly learned information may vary as a function of perceptual fluency and motor fluency.

## Introduction

Implicit learning (IL) is learning that occurs unconsciously, unintentionally, and without having declarative knowledge about what has been learned [1,2]. IL remains controversial in two ways. First, no agreement on the precise components of IL has been reached [3]. Second, there is some debate over how much control one can have over implicitly acquired knowledge before learning should be considered explicit rather than implicit (e.g., [4,5,6]). The present paper investigates the contribution of conscious and unconscious processes in the IL of temporal patterns using a recognition task based on the *process dissociation procedure* [7] and a model-based analysis adapted from a model by Buchner and colleagues [8,9,10]. Using previously unreported (post-test) recognition data from two experiments that demonstrated the IL of temporal patterns [11], we investigated the extent of conscious control in temporal pattern recognition and the role of fluency-based processes. The primary aim of the present study is to examine whether implicitly learned temporal patterns can be recognized via conscious or unconscious processes, and to test whether recognition via unconscious processes is associated with IL.

### The Process Dissociation Procedure

It is methodologically difficult to disentangle implicit and explicit processes while avoiding concerns of *process purity* [7,12]. Process purity is the assumption that performance in a task reflects a single process. However, this assumption is problematic when it is likely that both conscious and unconscious processes are engaged [13]. The process dissociation procedure [7] is a measure of IL that avoids concerns of process purity by using the same task under two types of instruction. For example, Karabanov and Ullén [14] used a generation task where the inclusion instruction required the reproduction of a learned temporal pattern and the exclusion instruction required the creation of novel temporal patterns. In the inclusion instruction, it is assumed that responses are facilitated by both implicit and explicit processes. In the exclusion instruction, it is assumed that explicit processes aid the suppression of the learned pattern but that implicit processes hinder the suppression of the learned pattern, at least partially due to motor fluency. Motor fluency is the speeded and/or relatively automatic response to a learned stimulus or pattern [15]. By comparing performance under inclusion and exclusion instructions, it is possible to ascertain the degree to which learned information is available to intentional control and, hence, whether learning was implicit [14,16,17]. That is, if the learned pattern is not able to be consciously reproduced, then the learned pattern would be generated under the exclusion instruction with similar or greater accuracy to that explicitly generated under the inclusion instruction, indicating that learning is implicit [14].

The IL of patterns of visual spatial locations [16,17] and auditory temporal patterns [11,14] has been demonstrated in generation tasks based on the process dissociation procedure. Furthermore, the generation task has been shown to be a sensitive test of implicit and explicit pattern knowledge [18]. In two experiments by Schultz et al. [11], IL was investigated using a serial reaction-time task (SRT), a generation task, and a recognition task based on the process dissociation procedure. Recognition data were not reported in the previous study as they were designed for the model-based analysis that is described in more detail here. The present paper reports data from the recognition task based on the process dissociation procedure where IL of auditory temporal patterns had been demonstrated in the SRT and the generation task referred to above.

### The Recognition Task

Recognition tasks based on the process dissociation procedure have been used to investigate whether participants can consciously recognize learned sequences [7,16,17]. In recognition tasks, participants are presented with a number of sequences or sequence fragments, some of which are the original acquisition pattern (i.e., the learned sequence) and some of which are novel sequences. Participants are asked to indicate whether they recognize the sequences or sequence fragments. If participants are able to identify the acquisition pattern and reject the novel sequences above the levels expected by chance, then they are said to have explicit knowledge of the sequence. The process by which one consciously recognizes a sequence is called *recollection* [19]. If participants are unable to show conscious recollection but have demonstrated learning in some other task (e.g., the SRT), then they are said to have implicitly learned the sequence [16,17]. *Familiarity* is proposed to refer to “automatic influences of memory” [19, pp. 665] and is assumed to be associated with unconscious knowledge. Although familiarity itself is a conscious feeling [20], the knowledge of the sequence that leads to the feeling of familiarity may still be conscious or unconscious [21,22].

A criticism of recognition tasks using the process dissociation procedure is that recollection and familiarity are positively correlated [23,24,25] and that the process dissociation procedure is not sensitive to this relationship. A stimulus may be correctly judged as old based on recollection or familiarity based on the following rationale: The behavior that demonstrates recollection is the correct identification of a stimulus that has previously been experienced [7]. However, the correct identification of a previously experienced stimulus can also be made based on familiarity. Familiarity occurs when a stimulus is judged as previously experienced due to similarity of stimulus features regardless of whether the stimulus has, in fact, been experienced [7]. Thus, familiarity occurs when a previously experienced stimulus is correctly judged as “old” due to recollection, *correctly* judged as “old” due to the similarity of stimulus features, or when a novel stimulus is *incorrectly* judged as “old” due the similarity of stimulus features. For example, the nursery rhymes “Twinkle twinkle, little star” and the “Alphabet song” have the same melody but different lyrics. If someone who had never heard either nursery rhyme were taught “Twinkle twinkle, little star”, then presented with the “Alphabet song” in a recognition task, they might recognize the melody feature but not truly recollect the “Alphabet song” that differs in regards to the lyrics. Thus, if asked if they were originally presented with the “Alphabet song”, they might incorrectly judge the stimulus as “old” based on familiarity with features of the melody. Similarly, they could make a correct recognition judgment of “Twinkle twinkle, little star” solely based on the melody, without any knowledge of the lyrics (i.e., without recollection). True recollection of the original nursery rhyme would consist of recognition of both the lyrics and melody and not mere familiarity with the nursery rhyme features. The relationship between recollection and familiarity is a concern when using recognition tasks based on the process dissociation procedure because responses indicating recollection may actually be attributable to familiarity of sequence features as opposed to recollection of the learned acquisition sequence.

A sense of familiarity with a stimulus in the absence of recollection or conscious identification of sequence features can also be elicited by perceptual fluency [7,19,26,27]. Perceptual fluency is the ease with which previously perceived features are processed [7], even if the object that possesses those features is novel. Perceptual fluency allows the correct identification of a previously experienced stimulus without the ability to make accurate judgments regarding why the object is familiar [7], that is, without conscious recollection of the object or the object features. Thus, perceptual fluency often (but not always; see 28,29) results in a sense of familiarity without conscious recollection [19,30]. In regards to sequence learning, the accurate discrimination of sequences that do or do not follow the rules of a grammar, often in the absence of the ability to explicitly state the rules, has been used to infer IL in artificial grammar learning paradigms (e.g., [21,22,31,32]). As discrimination in artificial grammar learning paradigms occurs due to familiarity with the grammatical features without recollection of the grammatical features, it is often assumed that familiarity without true recollection (i.e., perceptual fluency) can be used to infer IL (e.g., [8,9,10]). A similar concept is the *remember-know* distinction, where the *remember* aspect refers to conscious recollection, and the *know* aspect refers to familiarity that, when disentangled from conscious recollection, may also involve perceptual fluency [26]. The sequence identification measurement model (SIMM) [8,9,10] has been proposed as a computational method for separating the familiarity of sequence features (e.g., statistical regularities) from conscious recollection and unconscious recognition (via perceptual fluency).

### The Sequence Identification Measurement Model (SIMM)

Buchner and colleagues [8,9,10] used the recognition task based on the process dissociation procedure for the purpose of a model-based analysis referred to as the sequence identification measurement model (SIMM). In the recognition task based on the process dissociation procedure, participants are asked to respond to sequences that are identical to those learned, contain similar features to those learned, or contain different features to those learned. The features that were manipulated were the *systematicities* [8,9,10], that is, statistical regularities within a sequence, sequence fragments, and associative relationships between items within a sequence. A sequence could be considered systematic if the patterning of events within the sequence follows a set of rules. The inclusion instruction asks participants to respond “Yes” if the sequence is identical or contains similar features to the learned sequence and “No” if the sequence does not contain similar features to the learned sequence or appears unstructured. The exclusion instruction asks participants to respond “Yes” if the sequence contains similar features but is not identical to the learned sequence and “No” if the sequence is identical to the learned sequence or if it appears unstructured. The SIMM uses probability-based multinomial processing trees [33,34] on response frequencies to separate processes relating to the conscious recollection of the learned sequence, detection of systematicity, and detection of a lack of structure to extract parameters for conscious recollection (explicit) and unconscious recognition (implicit) that are not contaminated by familiarity with the sequence features. Importantly, the measure of unconscious processes in the SIMM is a more pure measure of perceptual fluency: perceptual fluency is disentangled from the familiarity of sequence features by estimating the influence of perceptual fluency separately from the influence of familiarity of the sequence features identified in the present sequences (see Appendix A in Text S1). The primary parameters of interest in the present study are those that refer to conscious and unconscious processes. The original SIMM has been successfully evaluated in a series of experiments [8,10] using the recognition task based on the process dissociation procedure.

In the SIMM, the parameter reflecting unconscious processes only represents IL under the assumption that perceptual fluency is related to, or can be used to infer, IL. The assumption that perceptual fluency is related to unconscious processing and IL was proposed by Buchner et al. [8]. However, Shanks and Johnstone [35] argue that perceptual fluency should not be viewed as related to IL because fluency may be experienced consciously. Furthermore, perceptual fluency may instead be an index of the level of conscious control one has over implicitly learned information [5]. For example, some experiments [36,37] have demonstrated that participants can correctly choose which of two artificial grammars to use in a given situation, despite reporting that they are guessing. This indicates that, although an individual may be able to recognize and use learned information (via conscious recollection, familiarity with the grammar, or perceptual fluency), they may not be able to identify how or why they are able to do so. Thus, it is possible that recollection or recognition via perceptual fluency can occur without awareness that learning has occurred, that is, learning was unintentional and, possibly, implicit.

Generation tasks are less affected by perceptual fluency because responses cannot be based on familiarity, that is, participants are not given a stimulus and, subsequently, cannot use features of a stimulus to make recognition judgments. Instead, the reproduction of the pattern can only be based on pattern knowledge and motor fluency. Thus, the generation task is not subject to the criticisms of the recognition task. A generation task based on the process dissociation procedure demonstrated that learning in Schultz et al. [11] was implicit. The recognition task and SIMM reported here were used to investigate IL under the assumption that perceptual fluency is experienced unconsciously.

### Hypotheses

The goal of the present study is to examine whether implicitly learned temporal sequences can be recognized via perceptual fluency and, in turn, to test whether perceptual fluency is associated with IL. The generation task in Schultz et al. [11] revealed IL of temporal patterns in Experiments 1 and 2. Thus, it is expected that the SIMM will demonstrate a greater contribution of unconscious (i.e., implicit) processes than conscious (i.e., explicit) processes to recognition judgments for Experiments 1 and 2. However, the SIMM is a measure of familiarity-based recognition and, as such, it can only be used to infer IL insofar as the premise that perceptual fluency reflects unconscious processes is true [8,10]. If the SIMM shows that unconscious processes play a greater role than conscious processes in the recognition of sequences, then it is likely that perceptual fluency is related to IL, as this result is congruent with those of Schultz et al. [11] that were obtained in a generation task. Alternatively, if the SIMM shows that conscious processes contribute to recognition judgments more than unconscious processes, then it is possible that perceptual fluency is not related to IL, as this result conflicts with the result of Schultz et al. [11]. In this way, a comparison of the results of the SIMM (based on a recognition task) and the outcomes of the generation task can be used to examine whether perceptual fluency (in the SIMM) occurs concurrently with IL (as demonstrated in the generation task).

Perceptual fluency is computed in the SIMM (applied to recognition data) by obtaining probability estimates for unconscious processes that are above zero and above the probability estimates for conscious processes [8,10]. If probability estimates for conscious processes are above zero and above the probability estimates of unconscious processes, then this would show explicit recollection of the learned pattern suggesting that learning was not implicit. If probability estimates for conscious and unconscious processes do not differ and are both significantly greater than zero, then the SIMM is unable to confirm or negate IL (insofar as IL is related to perceptual fluency), and learning may be viewed as partly implicit and partly explicit. If both conscious and unconscious parameter estimates are greater than zero, but one is greater than the other, then it is likely that both conscious and unconscious processes are involved, but one process is more involved than the other.

### Report of the Previous Study on the Implicit Learning of Temporal Patterns

In Schultz et al. [11], an SRT was used to investigate the IL of auditory temporal patterns. In the SRT, participants are presented with sequential stimuli and are asked to respond to each stimulus as quickly and accurately as possible [38]. Learning is characterized by: 1) a decrease in RT over blocks containing the repeating pattern, 2) RT increases when novel patterns are introduced, and 3) recovery of RT to previous latencies when the original acquisition pattern is reintroduced.

**Figure 1:**
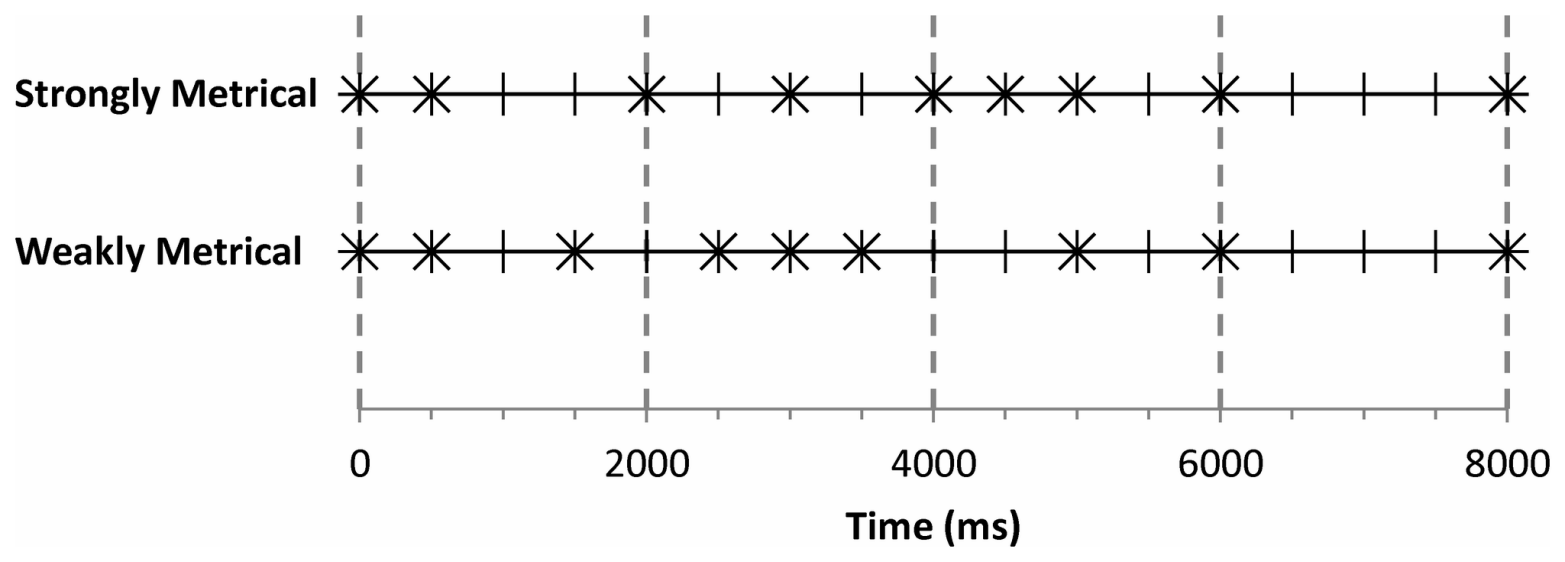
Beats (short vertical lines), strong beats (long vertical lines), and events (crosses) of the strongly metrical and weakly metrical temporal patterns. Beats and strong beats are a hypothetical cognitive framework and are not part of the stimulus itself.

The temporal patterns used in Schultz et al. [11] were patterns of inter-onset intervals (IOI) that are characteristic of musical rhythms (as in [39]). Rhythm is the “systematic patterning of sound in terms of timing, accent, and grouping” [40, pp. 96]. Meter is the sense of an isochronous pulse (or *beat*) that can be abstracted from a musical rhythm. Furthermore, the pulses are interpreted as alternating between strong and weak beats to form a hierarchical framework based on periodic timings [41,42]. Examples of rhythms, the *beat*, and first pulse of a group (*strong beats*) are given in Figure 1. Two types of rhythms were used: strongly metrical and weakly metrical [39,43]. A strongly metrical pattern contains events that occur on the *beat* and each *strong beat* contains an event. A weakly metrical pattern contains events that occur on the *beat* but do not always occur on the *strong beat*. It is possible that the metrical strength of a temporal pattern is a feature that is used when assessing whether a pattern is recollected or familiar.

Schultz et al. [11] examined IL of strongly metrical patterns in two experiments. Although the two conditions reported here were identical, the aim of the two experiments differed (see 11). In Experiment 1, IL of strongly metrical in a multiple response task was compared to IL in a single response task (i.e., both were adaptations of the classical SRT) with task type as a between-subjects variable. In Experiment 2, the IL of strongly metrical and nonmetrical patterns was investigated using a single response task, with metricality as a between-subjects variable. The present study examines recognition data from the strongly metrical conditions using the single response task in Experiments 1 and 2. In Experiments 1 and 2, results of the SRT demonstrated that metrical patterns were learned. There was also evidence that the metrical framework was learned in both experiments, as the introduction of a novel weakly metrical pattern resulted in a greater RT increase than the introduction of a novel strongly metrical pattern. Importantly, differences in RT increases to strongly and weakly metrical patterns demonstrated that people are sensitive to the feature of metrical strength [11]. Thus, it is imperative that models of the recognition of temporal patterns are sensitive to the detection of metrical frameworks and metrical strength. To ascertain whether learning was implicit, a generation task (based on [14]) was employed following the SRT. Results of the generation task in Experiments 1 and 2 revealed IL in the absence of familiarity and perceptual fluency-based cues and demonstrated that learning was implicit. After the generation task, participants performed the recognition task described in the present paper.

## Method

### Participants

Participants were first year Psychology students from the University of Western, Sydney. In Experiment 1, participants (*N* = 25; 21 female) had a mean age of 23.24 years (*SD* = 6.89, range 17-45). In Experiment 2, participants (*N* = 25; 12 female) had a mean age of 22.16 years (*SD* = 8.36, range 17-54). Participants in Experiment 2 had not participated in Experiment 1. No participant reported a hearing impairment.

### Stimuli

In the training blocks of the SRT, the stimuli could emanate from the left headphone, the right headphone, or both headphones in accordance with the cover story of a computer game for the blind (see 11). The cover story was implemented to reduce awareness of the temporal pattern in the SRT. In the recognition task, all stimuli were presented through both headphones (i.e., binaurally). The stimulus was a 394Hz triangle waveform of 200ms duration with 10ms rise and fall times. Stimuli were created using MAX-MSP and were presented using PsyScope software [44] through Sennheiser (HD 650) headphones.

**Figure 2:**
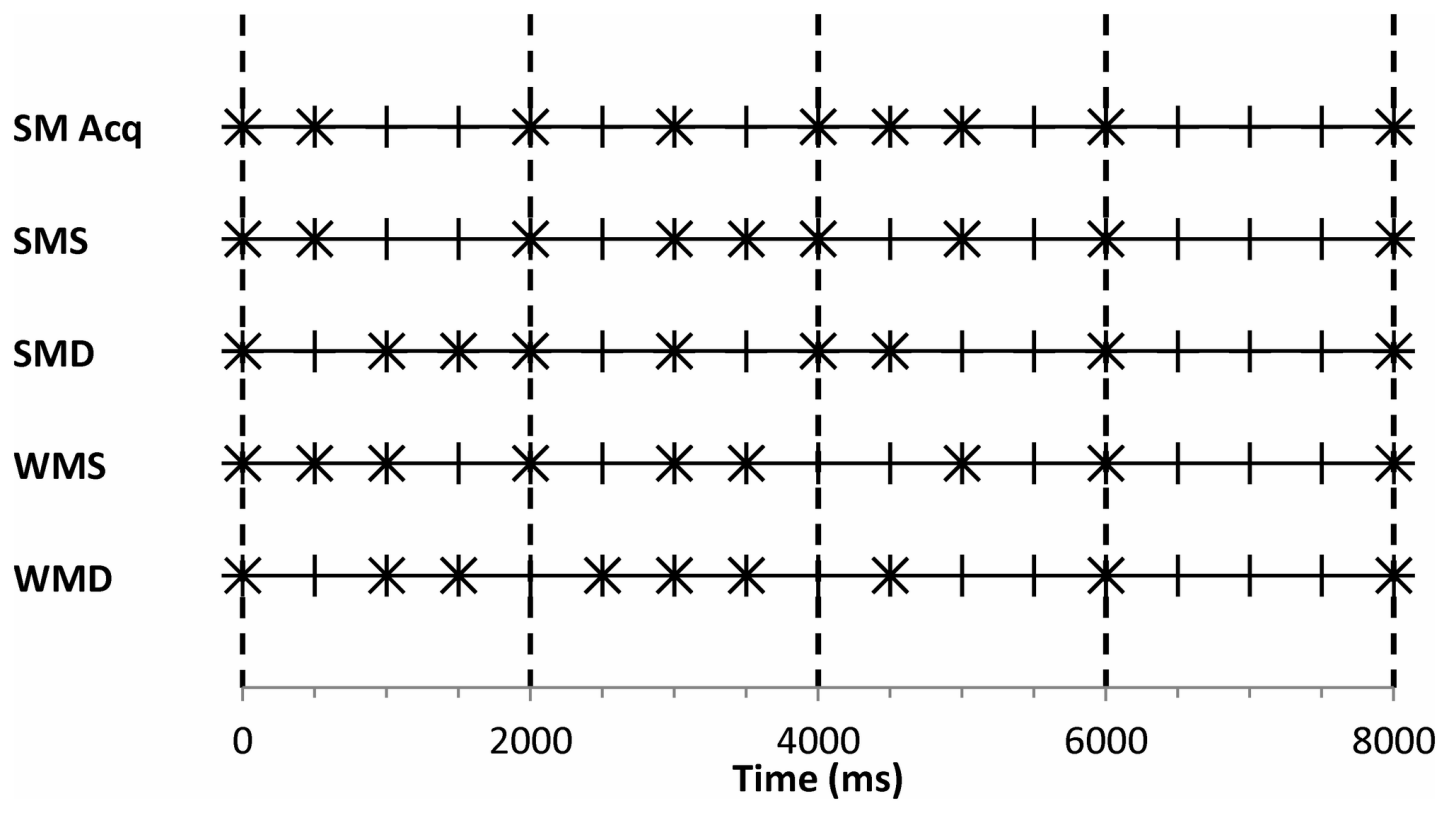
Metrical temporal patterns used in the recognition task. Events are represented by crosses, beats are represented by short vertical lines, and strong beats are represented by long dashed vertical lines. Sequences were: strongly metrical acquisition (SM Acq), strongly metrical systematic (SMS), strongly metrical distracter (SMD), weakly metrical systematic (WMS), and weakly metrical distracter (WMD).

The strongly metrical acquisition pattern used in the training blocks and the four sequences in the recognition task in the two experiments are shown in Figure 2. For example, the acquisition pattern in Figure 2 displays an IOI sequence (in ms) of 500-1500-1000-1000-500-500-1000-2000. All patterns consisted of three 500ms IOIs, three 1000ms IOIs, one 1500ms IOI, and one 2000ms IOI. In the recognition task, the acquisition pattern had been previously encountered in the SRT, and other four patterns were novel patterns (i.e., different from those presented in SRT training and test blocks).

Parameter estimates in the SIMM are calculated by taking into account which features of a sequence (e.g., statistical regularities and, in our case, also metrical strength) might be used to make recognition judgments and how these features are weighted. For this reason, temporal patterns in the recognition task must encompass and implement all possible combinations of features. The acquisition pattern has two features that may be learned: the statistical systematicities and the metrical structure. The statistical systematicities that we refer to here are the *simple frequency information* as outlined by Reed and Johnson [45]. Simple frequency information refers to statistical features of patterns that follow second order conditional probabilities, that is, a statistical property where each item can be predicted based on the two items that preceded it. For example, consider the pattern 1-3-2-2-1-1-2-4 where items 1, 2, 3, and 4 represent different temporal intervals. The pair 1-3 is always followed by 2, 3-2 is always followed by 2, 2-2 is always followed by 1, 2-1 is always followed by 1, 1-1 is always by 2, 1-2 is always followed by 4, and 2-4 is always followed by 1. After exposure to such a sequence, one would be able to predict the length of a temporal interval in the sequence based on the two intervals that preceded it.

Temporal patterns in Schultz et al. [11] were governed by second order conditional probabilities. For this reason, it is possible that participants were able to learn not only the second order conditional probabilities, but also the statistical structure called simple frequency information [45]. Simple frequency information refers to: the item frequency (the number of times an IOI occurs in a sequence), transition frequency (the number of times bigrams of items occur), the rate of full coverage (the average number of items that must occur to view each unique IOI in the sequence at least once), and the rate of full transition usage (the average number of items necessary to view each bigram at least once).

To use the SIMM to measure the influence of statistical features (i.e., simple frequency information) on recognition judgments, patterns with the same and different simple frequency information were presented. Novel systematic sequences in the recognition task had the same simple frequency information as the acquisition pattern. By contrast, novel distracter sequences in the recognition task had different simple frequency information than the acquisition pattern. Furthermore, to ensure that differences in responses in the recognition task cannot be attributed to recognition of (or familiarity with) temporal grouping differences, the size of rhythmic groupings (i.e., groups of two or three proximal events) was kept constant between patterns in the acquisition phase and the recognition phase.

To measure the influence of metrical features on recognition judgments (via the SIMM), metrical strength (i.e., strongly metrical, weakly metrical) was manipulated for novel sequences in the recognition task. Novel strongly metrical patterns had the same metrical strength as the acquisition pattern, and novel weakly metrical patterns had a weaker metrical strength than the acquisition pattern. The original SIMM of Buchner and colleagues [8,9,10] was used for nonmetrical temporal patterns [9] and, consequently, was not concerned with metrical features that specifically pertain to metrical temporal patterns. Hence, we adapted the SIMM to include a parameter that represents the conscious detection of metrical strength. The recognition task consisted of five sequences (see Figure 2): the strongly metrical acquisition (SM Acq) pattern, a strongly metrical systematic (SMS) sequence, a strongly metrical distracter (SMD) sequence, a weakly metrical systematic (WMS) sequence, and a weakly metrical distracter (WMD) sequence.

### Ethics Statement

Written informed consent was obtained from all participants and the study was approved by the University of Western Sydney Human Ethics Research committee (approval number H7764).

### Procedure

For a full description of the SRT and generation task, see Schultz et al. [11]. The recognition task was performed after the SRT and the generation task had been completed. In the recognition task, patterns were presented in a random order within each instruction (inclusion and exclusion). The order of inclusion and exclusion instruction was counterbalanced across participants. Participants were presented with each sequence and were asked to provide one “Yes” or “No” response per sequence in a binary decision task. The inclusion instruction was “Respond ‘Yes’ if the sequence was identical or had a similar structure to the sequence in the [SRT]” (

“SRT” here was replaced with “computer game for the blind” in line with the cover story used to promote IL, see [11] for details). The inclusion instruction required participants to respond “Yes” if the rhythmic pattern was identical to the one presented in the SRT or if it had familiar sequence features to the one presented in the SRT. Participants were asked to respond “No” if the pattern had unfamiliar sequence features. In this way, a “Yes” response in the inclusion instruction could reflect conscious recollection or perceptual fluency, or the familiarity with sequence features such as statistical systematicity or metrical strength. The exclusion instruction was “Respond ‘Yes’ if the sequence had a similar, but NOT identical, structure to the sequence in the [SRT].” The exclusion instruction required participants to only respond “Yes” if the sequence had familiar sequence features to the one presented in the SRT but was NOT identical to the one presented in the SRT. If participants recognized the pattern from the SRT, or if the pattern appeared to have unfamiliar sequence features, they were to respond “No”. In this way, a “Yes” response in the exclusion instruction reflects familiarity with the sequence features or perceptual fluency, but not conscious recollection of the acquisition pattern. A “No” response in the exclusion condition reflects conscious recollect

ion of the learned sequence.

**Figure 3:**
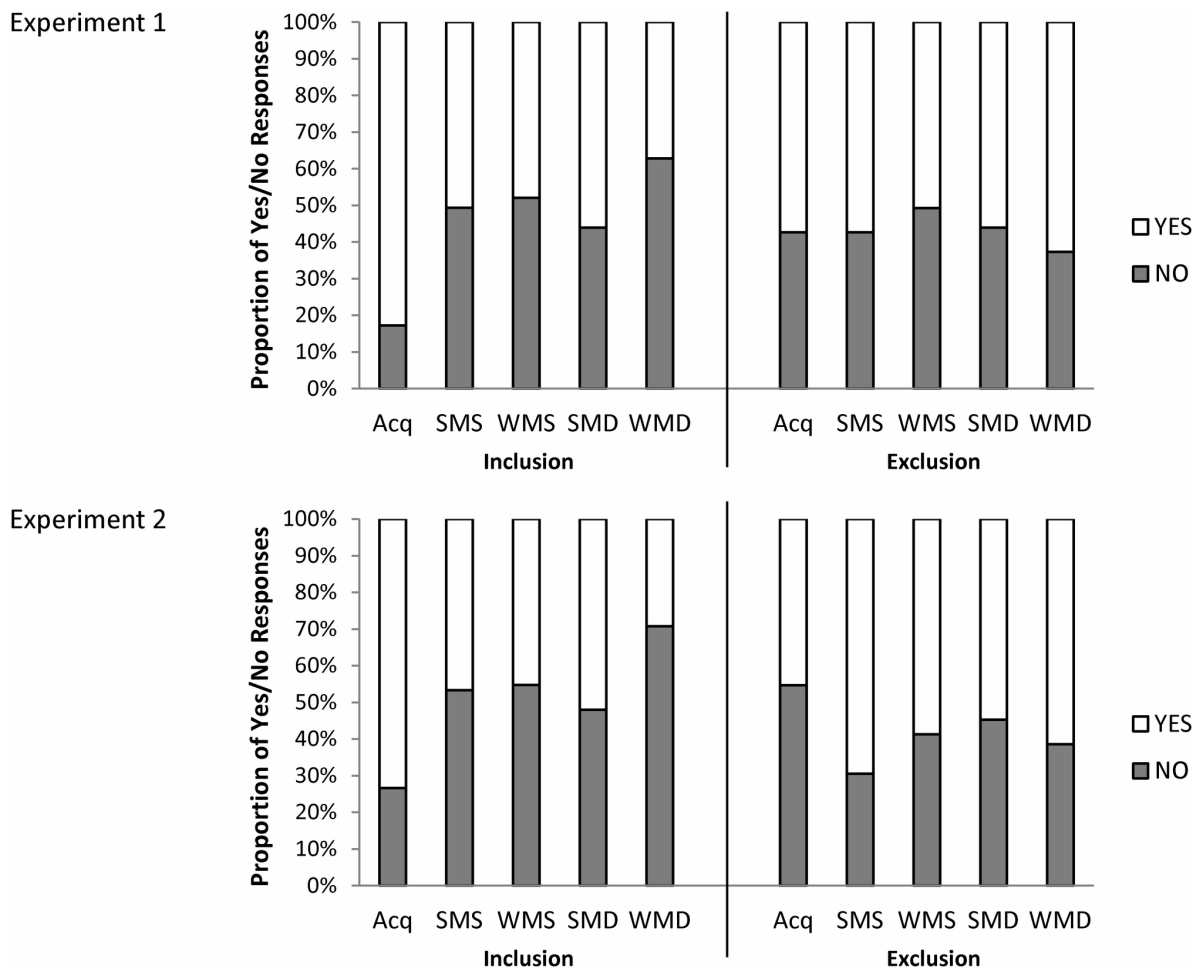
Behavioral data in the recognition task. Proportions of “Yes” (white bars) and “No” (grey bars) responses in the recognition task in the metrical conditions for Experiment 1 (top panel) and Experiment 2 (bottom panel). The temporal patterns presented under the inclusion and exclusion instruction were: the acquisition (Acq) sequence, the strongly metrical systematic (SMS) sequence, the weakly metrical systematic (WMS) sequence, the strongly metrical distracter (SMD) sequence, and the weakly metrical distracter (WMD) sequence.

Under each instruction, responses to the five sequences can be either “Yes” or “No”. In total, 20 different sets of responses can be made: 2 (instruction) x 5 (sequences) x 2 (answer, “Yes”/” No”), and each response corresponds to a different outcome in the processing tree (see Appendix A in Text S1, and Figure S1). Participants were presented with each sequence three times and the order of sequence presentations was random. In total, 60 responses were made per participant in the recognition task. The combined frequencies of responses from all participants were used to calculate the probabilities of the latent variables using the multinomial processing tree. The SIMM calculates the parameter estimates based on the pooled data of participants and, as such, parameter estimates reflect the contribution of processes at the group level, rather than the individual level. The proportion of “Yes” and “No” responses in Experiments 1 and 2 are shown in Figure 3 (see Appendix B in Text S2 for statistical analyses on the average proportions for each condition). These proportions are used in the SIMM to obtain parameter estimates. As can be seen in Figure 3, the relative proportions for the inclusion instruction were similar for Experiments 1 and 2. In the exclusion condition, however, the relative proportions differed; there were more “Yes” responses for the non-acquisition sequences compared to the acquisition sequence in Experiment 2. Thus, it was likely that the model outcome would differ between the two experiments.

### The Sequence Identification Measurement Model

Both the SIMM and the process dissociation procedure [7] share the assumption that participants respond differently under inclusion and exclusion instructions when a sequence is identified implicitly or explicitly. As mentioned, the original SIMM [8,9,10] did not consider temporal patterns comprised of IOIs and how rhythmic features, such as meter, might be learned and used to make recognition judgments. Due to the additional information given by meter and metrical strength, the original SIMM and parameter calculations needed to be modified accordingly. Here, we included the parameter *m* that represented the conscious detection of meter and/or metrical strength. A description of the SIMM and the calculations is presented in Appendix A (see Text S1).

The SIMM [8,9,10] as adapted here was used to analyze responses in inclusion and exclusion instructions and determine the value of the parameters reflecting conscious processes (*c*), unconscious processes (*uc-*

), detection of systematicity (*s*), detection of metrical strength (*m*), detection of a lack of structure (*d*), and guessing under the inclusion (*g_i_*) and exclusion (*g_e_*) instructions. Table 1 summarizes the parameters used in the present study. Although all parameters were included in the current study, based on previous implementations of the SIMM [8,9,10] it was expected that parameter *s* would not be greater than zero and would not significantly contribute to the model. Furthermore, due to the probability of guessing in binary decision tasks being 0.5, and previous results indicating that the guessing parameters in the SIMM are generally not significantly different from 0.5 [8,9,10], it was expected that the probability of guessing in the inclusion and exclusion instructions would not be significantly different from 0.5.

**Table 1:** Summary of the sequence identification measure model parameters.

**Parameter**	**Description**
*c*	The probability of consciously recollecting the learned acquisition sequence
*uc-*	The probability of recognizing the learned acquisition sequence via perceptual fluency
*s*	The probability of detecting statistical systematicity of the acquisition sequence based on transitional probabilities
*m*	The probability of detecting the metrical strength of the acquisition sequence (strongly metrical or weakly metrical)
*d*	The probability of detecting the absence of statistical systematicity and metrical strength of the acquisition sequence
*g^i^*	The probability of guessing in the inclusion instruction
*g^e^*	The probability of guessing in the exclusion instruction

## Results

Recognition data from Schultz et al. [11] (data is available upon request to the corresponding author) were analyzed using multiTree software [46], designed specifically for the analysis of joint multinomial processing tree models such as the SIMM and the adapted SIMM. MultiTree can be used to estimate model parameters, calculate variability (e.g., confidence intervals and standard error), and goodness-of-fit statistics. The Δ Akaike Information Criterion (ΔAIC) was used to assess the model fit to the data. The ΔAIC is a measure of a model relative to the “best” model given the current parameters [47]. ΔAIC does not give a significance level but, instead, provides a measure of the strength of evidence supporting the model where: 0 < ΔAIC < 2 suggests substantial evidence for the model; 3 < ΔAIC < 7 suggests considerably less support, and ΔAIC > 10 indicates that the model is unlikely. Negative ΔAIC values reflect overdispersion in the data, that is, greater variability in the data set than would be expected given the statistical model. The lowest positive value of ΔAIC was used to assess whether there was substantial evidence for the model [48,49]. Model fits were considered significant if the *p* value of the *PD* ^λ^ goodness-of-fit statistic is less than .05. Reference to a parameter estimate as “different” from a value (or another parameter estimate) refers to whether the upper and lower confidence interval (CI) of the parameter estimate overlap with the upper and lower CI of the other parameter estimate. Bootstrapped CIs (20,000 samples) were obtained following the advice of Hu [50] who stated that the Fisher information matrix (the default in multiTree) is not the best approximation of the true variance-covariance matrix in the case of small sample sizes.

First, following Buchner et al. [8,9,10] the processing trees were doubled so that separate parameter estimates could be obtained for Experiments 1 and 2. This was done simply to ensure that both experiments were comparable and because, from the outset, there were no reasons to suspect that the results would differ between the two experiments. To ensure that doubling the processing trees did not affect the outcome, the model was also run separately for each experiment. The resulting parameter estimates did not differ from those reported in the following sections and model fits for each condition were generally significant (or not significant) under the same model restrictions as those reported here.

When the model was fitted to the data without any restrictions (i.e., all parameters were free) the model fit fell short of significance (*PD* ^λ^ = 10.16, *p* = .26), but there was decent evidence for the model (ΔAIC = 2.16). The results of the unrestricted model are shown in Table 2. To refine the model, several parameter restrictions were applied (following Buchner et al. [8,10]). Based on the expectation that guessing under inclusion and exclusion instruction occurs with a probability of 0.5 (chance level for guessing), and the results of the unrestricted model that suggested these values were not different from 0.5 for all conditions (see Table 2), the parameters representing guessing under inclusion (*g*_*i*_) and exclusion (*g*_*e*_) instruction were set to 0.5. This resulted in a significant model fit (*PD* ^λ^ = 22.00, *p* = .02), but there was less evidence for the model (ΔAIC = 6.00) than for the unrestricted model. Based on previous results of the SIMM [8,9,10] and the results (in both the unrestricted model, and when parameters reflecting guessing are set to 0.5) indicating that the parameter reflecting the detection of statistical systematicity is not different from zero, the parameter *s* was also set to zero. Setting a parameter to zero is equivalent to removing the parameter from the model. This resulted in a significant model fit (*PD* ^λ^ = 22.67, *p* = .01) and demonstrated decent evidence for the model (ΔAIC = 2.67). As the parameters reflecting detection of metrical strength (m) and detection of a lack of structure (d) were not significantly different from zero in the unrestricted model (see Table 2), we systematically fit the model to the data with the restriction that each of these parameters were equal to zero for Experiments 1 and 2 (as performed by [8,9,10] for parameter *s*). The primary parameters of interest (reflecting conscious and unconscious processes) were not restricted.

**Table 2:** Parameter estimates for the unrestricted model in Experiments 1 and 2.

**Parameter**	**Experiment 1**		**Experiment 2**	
	**Parameter Estimate**	**CI (lower-upper)**	**Parameter Estimate**	**CI (lower-upper)**
*c*	0.30	0.21-0.39	0.28	0.12-0.45
*uc-*	0.55	0.37-0.73	0.16	-0.21-0.53
*s*	0.00	-0.25-0.25	0.11	-0.17-0.40
*m*	0.08	-0.16-0.32	0.17	-0.10-0.43
*d*	0.05	-0.31-0.40	0.00	-0.48-0.48
*g^i^*	0.46	0.32-0.59	0.37	0.14-0.61
*g^e^*	0.53	0.34-0.71	0.55	0.36-0.74

**Figure 4:**
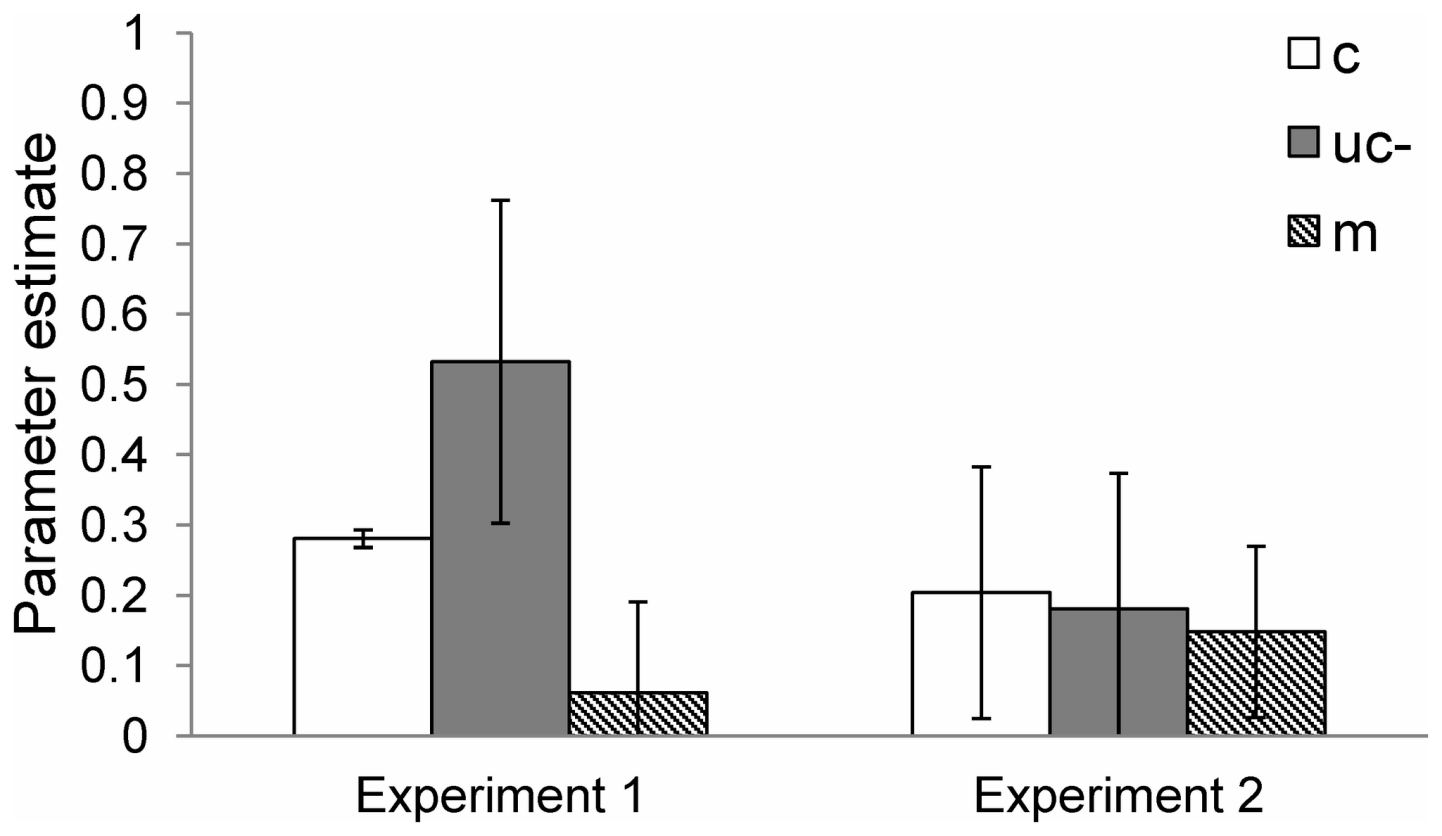
Parameter estimates for in Experiments 1 and 2. Probabilities range from 0 to 1. Parameters represent conscious processes (*c*; unfilled bars), unconscious processes (uc*-*; grey bars), and detection of the metrical strength (*m*; diagonally striped bars). Whiskers represent 95% confidence intervals (CI) estimated from 20,000 bootstrap samples. In Experiment 1, parameters *c* and *uc*- are considered different as the upper CI of parameter *c* (.29) does not overlap with the lower CI of parameter *uc*- (.30). In Experiment 2 parameters *c* and *uc*- are not considered different.

Of the three models tested, the model with the most substantial evidence (ΔAIC = 0.16, *PD* ^λ^ = 24.16, *p* = .049) resulted when the parameters reflecting the probability of guessing under inclusion (*g*_*i*_) and exclusion (*g*_*e*_) instruction were set to chance levels (0.5), and parameters reflecting the detection of systematicity (s) and the detection of a lack of structure (d) were set to zero. In effect, this reflects that the parameters of the detection of systematicity and the detection of a lack of structure were not notably contributing to the model. The resulting probability estimates for conscious processes (*c*), unconscious processes (*uc-*), and the detection of metrical strength (m) are shown in Figure 4.

The parameter representing conscious (c) processes was greater than zero for Experiment 1 and Experiment 2. Similarly, the parameter representing unconscious processes (*uc-*) was greater than zero for Experiments 1 and 2. As can be seen in Figure 4, for Experiment 1, unconscious processes (uc- = .53, CI = .30 to .76; indicative of perceptual fluency) contributed more to recognition judgments than conscious processes (c = .28, CI = .27 to .29). For Experiment 2, conscious (c = .20, CI = .02 to .38) and unconscious processes (uc- = .18, CI = -.01 to .48) contributed similarly to recognition judgments, indicating that responses were not primarily governed by perceptual fluency. Thus, there is a disagreement between the model-based evidence for perceptual fluency in Experiments 1 and 2. The parameter estimate for the detection of metrical strength was not significantly greater than zero in Experiment 1 (m = .06, CI = -.07 to .19) but was significantly greater than zero in Experiment 2 (m = .14, CI = .03 to .28). However, there was greater evidence for the model (as shown by ΔAIC) when this parameter was included for Experiments 1 and 2, indicating that the detection of metrical strength may still have contributed somewhat to recognition judgments. The model was also run on the pooled data from Experiments 1 and 2 (see Appendix C in Text S3), but the evidence for the model using the pooled data was not as strong (ΔAIC = 9.45, PD^λ^ = 21.45, p = .002) as the evidence found for the data with the two datasets separated (ΔAIC = 0.16, PD^λ^ = 24.16, p = .049) when the same restrictions were applied.

## Discussion

The present study used a model-based analysis to examine the contribution of conscious and unconscious processes to recognition judgments of temporal patterns that were learned in an SRT [11]. Results of the SIMM do not consistently support or refute the hypothesis that unconscious processes play a greater role in the recognition of temporal patterns than conscious processes. In Experiment 1, the SIMM indicated that both conscious and unconscious processes were involved, but that unconscious processes played a greater role than conscious processes. This finding is in line with the results of the generation task ( [11]: Experiment 1, single response condition) that demonstrated that learning was implicit. However, in Experiment 2, the SIMM indicated that both conscious and unconscious processes contributed equally to recognition judgments. Results of Experiment 2 are in contrast with the results of the generation task ( [11]: Experiment 2, metrical condition) that demonstrated that learning was implicit.

The discrepancy between the results of the recognition task (and SIMM) and the generation task might suggest a difference between perceptual fluency as shown in the recognition task and motor fluency as shown in the generation task. In other words, the discrepancy between the results of the recognition and generation tasks might be evidence that the assumption that perceptual fluency relates to IL does not always hold true, and that IL is better captured by the generation task that is not affected by familiarity-based processes. If we reject the assumption that perceptual fluency reflects IL, and instead treat perceptual fluency as a process that cannot be used to infer IL (as suggested by [35]), then we can conclude that learning of metrical patterns was implicit (as demonstrated in the generation task). However, it must be acknowledged that perceptual fluency may not have contributed to familiarity-based responses more strongly than conscious processes in Experiment 2 (as revealed by the recognition task and SIMM). In other words, unconscious processes contributed less to the recognition of patterns in Experiment 2 than in Experiment 1.

These results are not interpreted as evidence that the SIMM is invalid or unreliable: the SIMM has already been shown to be a reliable measure of perceptual fluency in a series of experiments [8,9,10]. Instead, we suggest that the SIMM parameter representing unconscious processes may not be associated with IL, but still measures perceptual fluency as per the assumptions of the model. One hypothesis is that, contrary to the assumptions of the SIMM [7,8,10], perceptual fluency is related to implicit knowledge and, as such, is a sufficient, but not a necessary, marker of IL. In other words, perceptual fluency might arise as a result of IL because the resulting knowledge is implicit, but a lack of perceptual fluency does not negate that the process of learning the sequences was implicit. Experimental conditions in Experiments 1 and 2 only differed with respect to the participants, and the results of the generation task demonstrated IL for both conditions. In contrast, the SIMM showed a greater influence of unconscious processes (via perceptual fluency) than conscious processes in Experiment 1, but not in Experiment 2. If perceptual fluency could be used to infer IL, then the results of the generation task and the SIMM should be congruent. The disagreement between the results of the generation task and the SIMM in Experiment 2 supports the hypothesis that perceptual fluency may not be a necessary requirement to conclude that learning is implicit.

It is also possible that the disagreement between the results of the generation task and the SIMM exposes differences in the amount of conscious control participants have over implicitly learned information. Some studies (e.g., [4,36,37]) have suggested that implicitly acquired information may still be available to conscious control (i.e., the information can be used strategically) even if the participant has no explicit knowledge of what has been learned and no intentional control over the use of knowledge that was implicitly learned. For example, Franco et al. [4] used an adaptation of a recognition task based on the process dissociation procedure to examine the degree of conscious control that could be exerted over knowledge that was acquired implicitly. Franco et al. demonstrated learning of two different artificial languages in the same participants. Results of Franco et al. showed that words from the languages could not be differentiated from one another, suggesting that learning was implicit. However, the words from the languages could be differentiated from new words, suggesting that participants could still exert conscious control over the implicitly learned information. In our present study, results of Experiment 2 are in line with the results of Franco et al. [4]: participants were able to differentiate the learned pattern from novel sequences in the recognition task, but could not create novel sequences that differed from the learned pattern in the generation task. These results suggest that, even though the patterns may have been implicitly learned, the learned information was still available to conscious control in a recognition task (in Experiment 2). Furthermore, Norman, Price, and Jones [51] hypothesize that there may be differences in the strategies and criteria used by individuals in recognition tasks. The use of different strategies or criteria might explain why conscious control differed between Experiments 1 and 2 presented here, that were similar in all respects other than participant sample.

Another possibility that is suggested by the disagreement between the generation task and the SIMM for Experiment 2 [11] is that there might be a difference between perceptual fluency and motor fluency. Perceptual fluency and motor fluency are sometimes discussed as interrelated processes (e.g., perceptual-motor fluency) in the SRT and implicit learning literature (e.g., [15,35]). It is possible that perceptual fluency and motor fluency represent different types of control that one has over the identification of sequences (i.e., perceptual fluency) and the recall or reproduction of sequences (i.e., motor fluency). As the recognition task primarily relies on perceptual influences, and the generation task primarily relies on motor influences, it is possible that the results from recognition and generation tasks in the present study have revealed that perceptual fluency and motor fluency may be dissociable.

Some evidence that perceptual and motor fluency are dissociable has already surfaced. A study by Gaillard, Destrebecqz, and Cleeremans [52] investigated the effects of increased attentional load during a generation post-test using visual spatial sequences. During the generation task, participants performed either an articulatory suppression task, a foot-tapping task, or no secondary task. Gaillard et al. found greater evidence for motor fluency in the exclusion task (i.e., an inability to suppress learned sequences) under conditions with a secondary task. Furthermore, participants could recognize sequence fragments above chance levels in a recognition task for all groups. This was viewed as evidence for a dissociation between conscious control in a generation task and recognition memory. Another interpretation that was not suggested by Gaillard et al. is that, in the generation task, the secondary task interfered with perceptual processes, resulting in more automatic motor responses. This interpretation would mean that, while perceptual fluency and motor fluency may be related, they may also play separate roles.

There is also evidence that motor fluency may affect perception. A study by Yang, Gallo, and Beilock [15] found that expert typists make more false recognition errors to perceived (not performed) letter dyads (i.e., non-words) that are considered more fluent to type, than those that are less fluent to type. This effect was reduced when a secondary finger-press motor task is performed during the recognition phase. Furthermore, novice typists did not exhibit fluency effects in the recognition task. This indicates that motor fluency may interfere with recognition judgments. Taken together, the results of Gaillard et al. [52] and Yang et al. [15] indicate that perceptual fluency and motor fluency have a complex association that begs investigation. If perceptual and motor fluency are dissociable, then this might explain the discrepancy between the generation task and the recognition task (and SIMM) in Experiments 1 and 2 in the present study. However, the role of perceptual fluency in implicit learning is still uncertain.

## Conclusion

The present study presented a model-based analysis for examining the IL of temporal patterns. The adapted SIMM developed here included a parameter for the detection of metrical strength, and results of the model demonstrated that the inclusion of this parameter improved the model. In tandem with the results of Schultz et al. [11], the model suggests that perceptual fluency may not necessarily be associated with IL. These results are in line with the conclusions of Shanks and Johnstone [35] that fluency can be experienced consciously. Alternatively, the model suggests differences in the amount of control that individuals have over implicitly learned information (as suggested by [4]), a speculation that cannot be confirmed in the present study, and requires further testing. A limitation is that the SIMM uses frequency data and was not designed to account for individual differences; the development of another model that can be used on individuals’ data would be a valuable avenue for future research that could test the hypothesis that there are individual differences regarding strategic control over implicitly learned information. Another interpretation of the present results is that perceptual and motor fluency could be dissociable processes. Future experiments examining perceptual and motor fluency under conditions of attentional load or with a secondary motor task are necessary to uncover how perceptual and motor fluency are related. As the results of Buchner and colleagues [8,9,10] have suggested that the SIMM is a measure of perceptual fluency, the SIMM might be useful for exploring the role of perceptual fluency in the recognition of sequences when perceptual or motor processes are engaged in a secondary task. However, the present results suggest that there may be an uncertain relationship between perceptual fluency and IL that requires further investigation.

## Supporting Information

Figure S1 **The adapted sequence identification measurement model for the inclusion and exclusion test conditions.** The sequence types are shown on the left, participants’ responses (“Yes” and “No”) are shown on the right, and the parameters denoting the probabilities with which the underlying cognitive states are arrived at constitute the middle. The parameters represent the probability of consciously recollecting the Acquisition pattern systematicity (parameter *c*), the probability of detecting the systematicity in a sequence that cannot be recollected (parameter *s*), the probability of detecting the metrical strength in a sequence that cannot be recollected (parameter *m*), the probability of recognizing the acquisition pattern via perceptual fluency (parameter *u*c-), the guessing that a sequence requires a “Yes” response in the absence of any other information about the sequence (parameters *g*_i_ and *g*_e_ in the inclusion and exclusion test conditions, respectively), and the detection of a lack of structure (parameter d).
(TIF)

Text S1 **Appendix A: Description of the Sequence Identification Measurement Model.**
(DOCX)

Text S2 **Appendix B: Statistical analyses on proportion data.**
(DOCX)

Text S3 **Appendix C: Analysis of pooled data from Experiments 1 and 2.**
(DOCX)
