# Supplementary figures and images for: A Sequence Identification Measurement Model to Investigate the Implicit Learning of Metrical Temporal Patterns
Source: PLoS One. 2013 Sep 25;8(9):e75163. doi: 10.1371/journal.pone.0075163 (PMC3783451; doi:10.1371/journal.pone.0075163)

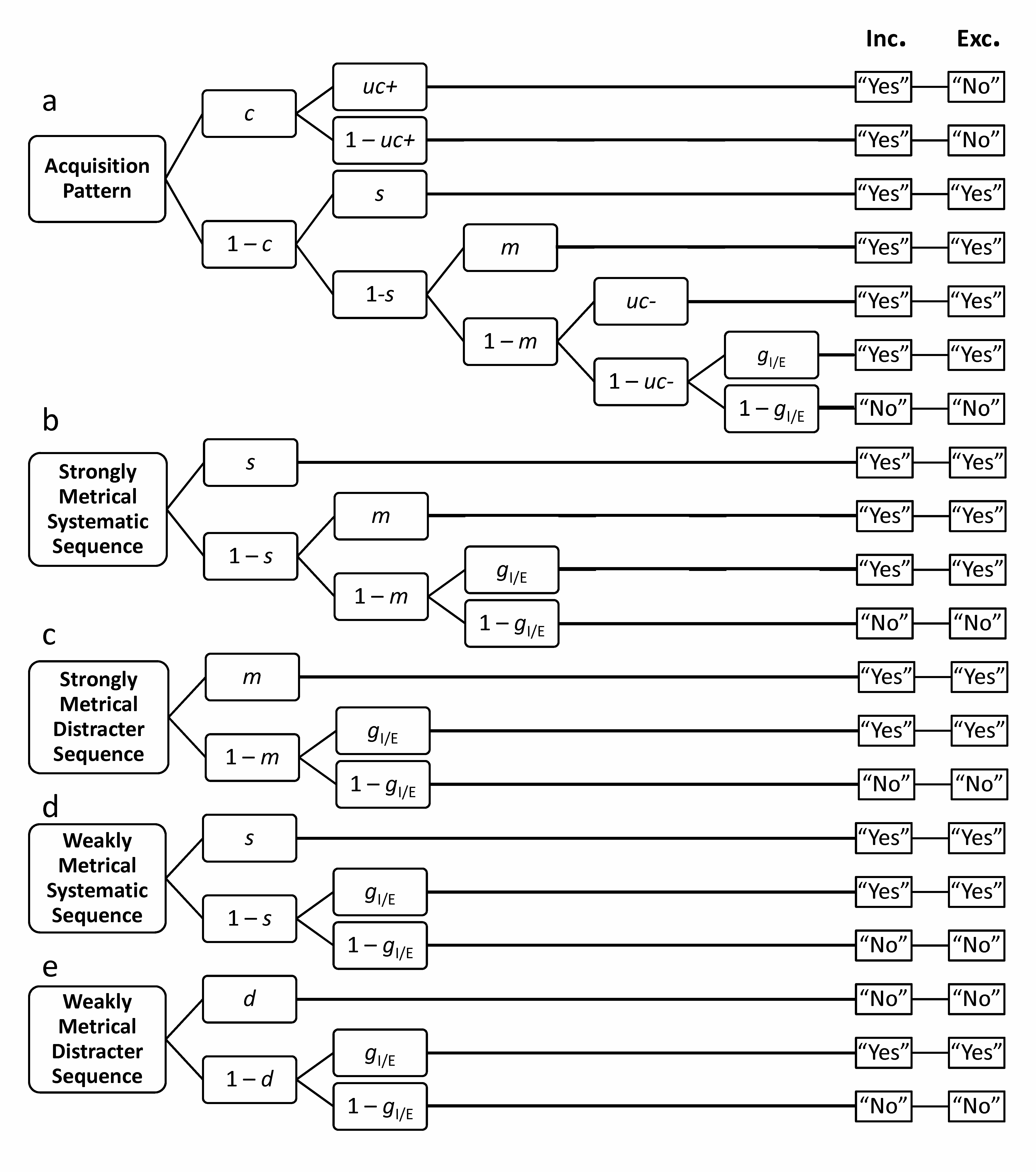

Supplement: Figure S1 — The adapted sequence identification measurement model for the inclusion and exclusion test conditions. The sequence types are shown on the left, participants’ responses (“Yes” and “No”) are shown on the right, and the parameters denoting the probabilities with which the underlying cognitive states are arrived at constitute the middle. The parameters represent the probability of consciously recollecting the Acquisition pattern systematicity (parameter c), the probability of detecting the systematicity in a sequence that cannot be recollected (parameter s), the probability of detecting the metrical strength in a sequence that cannot be recollected (parameter m), the probability of recognizing the acquisition pattern via perceptual fluency (parameter uc-), the guessing that a sequence requires a “Yes” response in the absence of any other information about the sequence (parameters g i and g e in the inclusion and exclusion test conditions, respectively), and the detection of a lack of structure (parameter d). (TIF) [file pone.0075163.s001.tif]
