# Supplementary material for: A Sequence Identification Measurement Model to Investigate the Implicit Learning of Metrical Temporal Patterns
Source: PLoS One. 2013 Sep 25;8(9):e75163. doi: 10.1371/journal.pone.0075163 (PMC3783451; doi:10.1371/journal.pone.0075163)
Supplement: Text S1 — Appendix A: Description of the Sequence Identification Measurement Model. (DOCX) [file pone.0075163.s002.docx]

**TEXT S1**

**Appendix A: Description of the Sequence Identification Measurement Model**

The processing trees in Figure S1 illustrate the probabilities for responses to the strongly metrical acquisition (ACQ) pattern, the strongly metrical systematic (SMS) sequence, the weakly metrical systematic (WMS) sequence, the strongly metrical distracter (SMD) sequence, and the weakly metrical distracter (WMD) sequence according to the modified SIMM [8, 9, 10]. Parameter *c* (representing conscious processes) is the conditional probability that participants show conscious recollection of the acquisition pattern. The probability of not recollecting an acquisition pattern is 1 - *c* (see the tree a in Figure S1). If the acquisition pattern is not consciously recollected, a recognition judgment may still be based on the conscious detection of sequence systematicity with the conditional probability *s*. If the acquisition pattern is not consciously recollected, and its systematicity is not detected, then a recognition judgment may still be based on the conscious detection of metrical strength with the conditional probability *m*. The detection of metrical strength is based on whether the sequence is considered strongly metrical with the same metrical framework as the acquisition pattern. If the sequence is not consciously recollected, and neither the systematicity nor the metrical strength is detected, the sequence might still receive a recognition judgment through perceptual fluency with the conditional probability *uc-* (reflecting unconscious processes). If the acquisition pattern is not consciously recollected, its systematicity is not detected, the metrical strength is not detected, and is not recognized via perceptual fluency, then participants may still make a recognition judgment based on a guess. Parameters *g_i_* and *g_e_* represent the conditional probability of guessing “Yes” under inclusion and exclusion instructions, respectively. The parameter *uc+* is the unconditional probability of unconscious processes affecting conscious recollection judgments, and is left unrestricted following Buchner at al. [8, 10]. The parameter *uc+* has been retained to keep in line with the original SIMM [8, 9, 10], but as *uc+* is left unrestricted, it does not affect the model and the parameter estimates.

Novel sequences cannot be consciously recollected or processed fluently in the same manner as the acquisition pattern. However, systematicity (*s*) and metrical strength (*m*) can be detected in novel sequences that contain these features. For the novel strongly metrical systematic sequence (see tree b in Figure S1) participants could detect either the systematicity (*s*) or the metrical strength (*m*). If systematicity is not detected (with a probability of 1 - *s*), then it is still be possible that participants detected the metrical strength (with a probability of *m*). If neither the systematicity nor metrical strength is detected, with a probability of
(1 - *s*) × (1 - *m*), then no other stimulus information is available and it is assumed that participants will guess (*g_i_* and *g_e_*). For the novel strongly metrical distracter sequence (see tree c in Figure S1), participants could detect the metrical strength with probability *m*. Otherwise, it is assumed that participants guess (*g_i_* and *g_e_*). For the novel weakly metrical systematic sequence (see tree d in Figure S1), participants could detect the systematicity with probability *s*. Otherwise, it is assumed that participants guess (*g_i_* and *g_e_*).

The novel weakly metrical distracter sequence cannot be recollected or processed fluently in the same way as the acquisition pattern. However, participants may still detect the lack of structure of a novel weakly metrical distracter sequence or a violation to statistical systematicity and metrical strength with conditional probability *d* (see tree e in Figure S1). If the lack of structure is not detected (with probability 1 - *d*), it is assumed that participants will guess (*g_i_* and *g_e_*). Based on the rationale outlined above and represented in the processing trees, one is able to derive probability estimates from the responses given in inclusion and exclusion conditions.

As in Buchner et al*.* [8, 10], an acquisition phase pattern may be consciously recollected with probability *c*. Now, the sequence may not be recollected but it may be identified as systematic with probability (1 - *c*) × *s*. If systematicity is not identified, metricality might still be detected with probability (1 - *c*) × (1 - *s*) × *m*. If neither systematicity nor metricality is identified, then the sequence may still be accepted as well-formed due to perceptual fluency with probability (1 - *c* ) × (1 - *s*) × (1 - *m*) × *u*c-. Finally, a “Yes” response may simply be the result of guessing with probability (1 - *c*) × (1 - *s*) ×
(1 - *m*) × (1 - *u*c-) × *g*_i_. Note that the order of *s* and *m* is inconsequential in the present design due to the inclusion of sequences that measure systematicity and metrical strength independently from one another. In other words, the multinomial processing trees implemented here exploited sequences that measured the influence of the detection of systematicity in isolation of the detection of metrical strength (the WMS sequence) and the detection of metrical strength in isolation of the detection of systematicity (the SMD sequence). To ensure that the order of *s* and *m* was inconsequential, the model was run with two different configurations of parameter *s* and parameter *m*: one where parameter *s* was assumed to be processed first, and parameter *m* was processed second, and another where parameter *m* was assumed to be processed first, and parameter *s* was processed second. Results were identical for the two configurations indicating that the order of *s* and *m* in the model did not affect the outcome.

From the multinomial processing tree, ten equations can be derived for the probability of responding “Yes” to the five sequences in the inclusion and exclusion instructions.

**Inclusion Instruction:**

Acquisition pattern (ACQi)

1. $\boldsymbol{ACQi}\boldsymbol{=}\boldsymbol{c}\boldsymbol{\times}\boldsymbol{u}_{\boldsymbol{c}\boldsymbol{+}}\boldsymbol{+}\boldsymbol{c}\boldsymbol{\times}\left( \boldsymbol{1}\boldsymbol{-}\boldsymbol{u}_{\boldsymbol{c}\boldsymbol{+}} \right)\boldsymbol{+}\left( \boldsymbol{1}\boldsymbol{-}\boldsymbol{c} \right)\boldsymbol{\times}\boldsymbol{s}\boldsymbol{+}\left( \boldsymbol{1}\boldsymbol{-}\boldsymbol{c} \right)\boldsymbol{\times}\boldsymbol{m}\boldsymbol{+}\left( \boldsymbol{1}\boldsymbol{-}\boldsymbol{c} \right)\boldsymbol{\times}\left( \boldsymbol{1}\boldsymbol{-}\boldsymbol{s} \right)\boldsymbol{\times}\left( \boldsymbol{1}\boldsymbol{-}\boldsymbol{m} \right)\boldsymbol{\times}\boldsymbol{u}_{\boldsymbol{c}\boldsymbol{-}}\boldsymbol{+}\left( \boldsymbol{1}\boldsymbol{-}\boldsymbol{c} \right)\boldsymbol{\times}\left( \boldsymbol{1}\boldsymbol{-}\boldsymbol{s} \right)\boldsymbol{\times}\left( \boldsymbol{1}\boldsymbol{-}\boldsymbol{m} \right)\boldsymbol{\times}\left( \boldsymbol{1}\boldsymbol{-}\boldsymbol{u}_{\boldsymbol{c}\boldsymbol{-}} \right)\boldsymbol{\times}\boldsymbol{g}_{\boldsymbol{i}}$

Strongly metrical systematic sequence (SMSi)

1. $\boldsymbol{SMSi}\boldsymbol{=}\boldsymbol{s}\boldsymbol{+}\left( \boldsymbol{1}\boldsymbol{-}\boldsymbol{s} \right)\boldsymbol{\times}\boldsymbol{m}\boldsymbol{+}\left( \boldsymbol{1}\boldsymbol{-}\boldsymbol{s} \right)\boldsymbol{\times}\left( \boldsymbol{1}\boldsymbol{-}\boldsymbol{m} \right)\boldsymbol{\times}\boldsymbol{g}_{\boldsymbol{i}}$

Weakly metrical systematic sequence (WMSi)

1. $\boldsymbol{WMSi}\boldsymbol{=}\boldsymbol{s}\boldsymbol{+}\left( \boldsymbol{1}\boldsymbol{-}\boldsymbol{s} \right)\boldsymbol{\times}\boldsymbol{g}_{\boldsymbol{i}}$

Strongly metrical distracter sequence (*SMD*i)

1. $\boldsymbol{SMDi}\boldsymbol{=}\boldsymbol{m}\boldsymbol{+}\left( \boldsymbol{1}\boldsymbol{-}\boldsymbol{m} \right)\boldsymbol{\times}\boldsymbol{g}_{\boldsymbol{i}}$

Weakly metrical distracter sequences (*WMD*i)

1. $\boldsymbol{WMDi}\boldsymbol{=}\left( \boldsymbol{1}\boldsymbol{-}\boldsymbol{d} \right)\boldsymbol{\times}\boldsymbol{g}_{\boldsymbol{i}}$

**Exclusion instruction:**

Acquisition pattern (*ACQe*)

1. $\boldsymbol{ACQe}\boldsymbol{=}\left( \boldsymbol{1}\boldsymbol{-}\boldsymbol{c} \right)\boldsymbol{\times}\boldsymbol{s}\boldsymbol{+}\left( \boldsymbol{1}\boldsymbol{-}\boldsymbol{c} \right)\boldsymbol{\times}\left( \boldsymbol{1}\boldsymbol{-}\boldsymbol{s} \right)\boldsymbol{\times}\boldsymbol{m}\boldsymbol{+}\left( \boldsymbol{1}\boldsymbol{-}\boldsymbol{c} \right)\boldsymbol{\times}\left( \boldsymbol{1}\boldsymbol{-}\boldsymbol{s} \right)\boldsymbol{\times}\left( \boldsymbol{1}\boldsymbol{-}\boldsymbol{m} \right)\boldsymbol{\times}\boldsymbol{u}_{\boldsymbol{c}\boldsymbol{-}}\boldsymbol{+}\left( \boldsymbol{1}\boldsymbol{-}\boldsymbol{c} \right)\boldsymbol{\times}\left( \boldsymbol{1}\boldsymbol{-}\boldsymbol{s} \right)\boldsymbol{\times}\left( \boldsymbol{1}\boldsymbol{-}\boldsymbol{m} \right)\boldsymbol{\times}\left( \boldsymbol{1}\boldsymbol{-}\boldsymbol{u}_{\boldsymbol{c}\boldsymbol{-}} \right)\boldsymbol{\times}\boldsymbol{g}_{\boldsymbol{e}}$

Strongly metrical systematic sequences (*SMS*e)

1. $\boldsymbol{SMSe}\boldsymbol{=}\boldsymbol{s}\boldsymbol{+}\left( \boldsymbol{1}\boldsymbol{-}\boldsymbol{s} \right)\boldsymbol{\times}\boldsymbol{m}\boldsymbol{+}\left( \boldsymbol{1}\boldsymbol{-}\boldsymbol{s} \right)\boldsymbol{\times}\left( \boldsymbol{1}\boldsymbol{-}\boldsymbol{m} \right)\boldsymbol{\times}\boldsymbol{g}_{\boldsymbol{e}}$

Weakly metrical systematic sequences (*WMS*e)

1. $\boldsymbol{WMSe}\boldsymbol{=}\boldsymbol{s}\boldsymbol{+}\left( \boldsymbol{1}\boldsymbol{-}\boldsymbol{s} \right)\boldsymbol{\times}\boldsymbol{g}_{\boldsymbol{e}}$

Strongly metrical distracter sequences (*SMD*e)

1. $\boldsymbol{SMDe}\boldsymbol{=}\boldsymbol{m}\boldsymbol{+}\left( \boldsymbol{1}\boldsymbol{-}\boldsymbol{m} \right)\boldsymbol{\times}\boldsymbol{g}_{\boldsymbol{e}}$

Weakly metrical distracter sequences (*WMD*e)

1. $\boldsymbol{WMDe}\boldsymbol{=}\left( \boldsymbol{1}\boldsymbol{-}\boldsymbol{d} \right)\boldsymbol{\times}\boldsymbol{g}_{\boldsymbol{e}}$
