# Supplementary material for: A Sequence Identification Measurement Model to Investigate the Implicit Learning of Metrical Temporal Patterns
Source: PLoS One. 2013 Sep 25;8(9):e75163. doi: 10.1371/journal.pone.0075163 (PMC3783451; doi:10.1371/journal.pone.0075163)
Supplement: Text S2 — Appendix B: Statistical analyses on proportion data. (DOCX) [file pone.0075163.s003.docx]

**TEXT S2**

**Appendix B: Statistical analyses on proportion data**

A repeated measures analysis of variance (ANOVA) was conducted on the average proportions for each participant in each instruction (inclusion, exclusion; within-subjects), for each sequence (Acquisition, SMS, WMS, SMD, WMD; within-subjects), in each experiment (Experiment 1, Experiment 2; between-subjects). There were significant main effects of instruction (*F* (1, 49) = 19.54, *p* < .001, *η_p_^2^* = .29) and sequence (*F* (4, 196) = 2.47, *p* = .046, *η_p_^2^* = .05), and a significant interaction between sequence and instruction (*F* (4, 196) = 8.66, *p* < .001, *η_p_^2^* = .15). No other effects were significant (*ps* > .43). In the inclusion instruction, pair-wise comparisons demonstrated that the Acquisition sequence was different from all other sequences (*ps* < .003), the WMS sequence was near-significantly different from the WMD sequence (*p* = .08), and the SMD sequence was significantly different from the WMD sequence (*p* = .01) (other pair-wise comparisons were not significant). In the exclusion instruction, the Acquisition sequence was significantly different from the SMS sequence (*p* < .001) and near-significantly different from the WMS sequence (*p* = .09), the SMS sequence was significantly different from the SMD sequence (*p* = .04) and was near-significantly different from the WMD sequence (*p* = .08), and other pair-wise comparisons were not significant. These results demonstrate that there were significant differences between responses to sequences, and that these differences were moderated by the instruction (i.e.,
inclusion, exclusion).
