# Supplementary material for: A Sequence Identification Measurement Model to Investigate the Implicit Learning of Metrical Temporal Patterns
Source: PLoS One. 2013 Sep 25;8(9):e75163. doi: 10.1371/journal.pone.0075163 (PMC3783451; doi:10.1371/journal.pone.0075163)
Supplement: Text S3 — Appendix C: Analysis of pooled data from Experiments 1 and 2. (DOCX) [file pone.0075163.s004.docx]

**TEXT S3**

**Appendix C: Analysis of pooled data from Experiments 1 and 2**

To examine whether a more parsimonious model could be ascertained by pooling the data from Experiments 1 and 2, the frequencies of “Yes” and “No” responses to each sequence under each instruction for all participants in Experiments1 and 2 were combined. When the model was fitted to the data without any restrictions (i.e., all parameters were free) the model fit was significant (*PD*^λ^ = 10.00, *p* = .04) and there was moderate evidence for the model (ΔAIC = 6.00). When the model was fit with the probability of guessing under inclusion and exclusion instructions set to0.5, the model was still significant (*PD*^λ^ = 19.95, *p* = .002) but there was evidence that the model did not fit the data (ΔAIC = 11.95). When parameter *s* was restricted to zero (following [8, 9, 10]), with all other parameters free (including guessing in the inclusion and exclusion conditions) the model showed a significant fit (*PD*^λ^ = 10.00, *p* = .045) and there was moderate evidence for the model (ΔAIC = 4.00). The best model fit from the unpooled data from Experiments 1 and 2 was obtained when parameters *d* and *s* were set to zero, and parameters reflecting guessing in the inclusion and exclusions conditions were set to 0.5. To compare the model fits of the pooled and unpooled data, the same restrictions were imposed on the pooled data. The model was significant (*PD*^λ^ = 21.45, *p* = .003) but there was evidence that the model did not fit the data (ΔAIC = 9.45). The model with the most evidence (ΔAIC = 2.77) was obtained when parameters d and s were restricted to zero and all other parameters were free, however, the model fell short of significance (*PD*^λ^ = 10.77, *p* = .06). The parameters reflecting conscious and unconscious processes were not significantly different from one another when the model showed substantial evidence and significant model fits. However, the parameter reflecting unconscious processes (*uc-* = .43, *CI* = .27 to .58) was larger than the parameter representing conscious processes (*c* = .24, *CI* = .24 to .25) when guessing parameters were set to0.5, and parameters reflecting the detection of systematicity and metrical strength were set to zero although the model did not demonstrate strong evidence (ΔAIC = 12.76; *PD*^λ^ = 24.76,
*p* < .001).

The results of the SIMM implementation with the pooled frequencies from Experiments 1 and 2 did not demonstrate a superior model fit to that produced when the data from Experiments 1 and 2 were separated. If the results obtained by pooling the data showed significant data fits with substantial evidence, then it could have been used to support or refute the hypothesis that perceptual fluency is equivalent to IL, and that the difference between the results obtained in Experiments 1 and 2 arose due to chance. That the model fits were better when data were separated provides some support that the differences between Experiments 1 and 2 reflect differences in the emergence of perceptual fluency under identical conditions where IL was observed.
